# COVID-19 Pandemic Quarantines and Mental Health Among Adolescents in Norway

**DOI:** 10.1001/jamanetworkopen.2024.22189

**Published:** 2024-07-12

**Authors:** Johanne H. Pettersen, Laurie J. Hannigan, Kristin Gustavson, Ingunn O. Lund, Rebecca M. Pearson, Pia Jensen, Ragnar Nesvåg, Ragnhild E. Brandlistuen, Helga Ask

**Affiliations:** 1 PsychGen Center for Genetic Epidemiology and Mental Health, Norwegian Institute of Public Health, Oslo, Norway; 2 Department of Child Health and Development, Norwegian Institute of Public Health, Oslo, Norway; 3 Department of Psychology, University of Oslo, Oslo, Norway; 4 Nic Waals Institute, Lovisenberg Diaconal Hospital, Oslo, Norway; 5 Population Health Sciences, Bristol Medical School, University of Bristol, Bristol, United Kingdom; 6 Department of Children and Families, Norwegian Institute of Public Health, Oslo, Norway; 7 Department of Psychology, Manchester Metropolitan University, Bristol, Untied Kingdom; 8 Division of Mental and Physical Health, Norwegian Institute of Public Health, Oslo, Norway; 9 The Norwegian Mother, Father, and Child Cohort Study (MoBa), Norwegian Institute of Public Health, Oslo, Norway

## Abstract

**Importance:**

Adolescence is a critical developmental phase when mental health disorders, such as anxiety and depression, often emerge. Stringent public health measures and quarantine mandates during the COVID-19 pandemic could threaten adolescent mental health.

**Objective:**

To investigate the associations of public health measures and quarantine experiences with mental distress among Norwegian adolescents and to explore if certain vulnerability factors moderate these associations.

**Design, Setting, and Participants:**

This longitudinal cohort study used repeated measures to capture variations in mental distress explained by the stringency of public health measures and quarantine experiences. Data from the Norwegian Mother, Father, and Child cohort study were linked to national health registries and a national stringency index from April 1, 2020, to February 17, 2021. Participant included 7787 Norwegian adolescents aged 16 to 18 years. Data were analyzed from October 2022 to October 2023.

**Exposures:**

Stringency index of public health measures and quarantine experiences including recent quarantine (within the last 2 weeks) and quarantine frequency (cumulative number of quarantine episodes).

**Main Outcome and Measures:**

Mental distress was measured using the Hopkins Symptom Checklist across 6 data collection waves.

**Results:**

In this study, 7787 participants were included in the analysis (4473 female [57%]; mean [SD] age, 17.0 [0.6] years). Stringent public health measures (β = 0.18; SE, 0.02; *P* < .001), recent quarantine (β = 0.11; SE, 0.02; *P* < .001), and frequent quarantine (β = 0.08; SE, 0.01; *P* < .001) were associated with higher levels of mental distress. The associations between public health measures and mental distress were not moderated by sex, age, prepandemic anxiety or depression, or genetic liability for mental health conditions. Frequency of quarantine appeared to be more strongly associated with mental distress among younger adolescents (β = −0.04; SE, 0.01; *P* = .008), those with parents with lower education (β = −0.04; SE, 0.01; *P* = .007), and those with lower genetic risk for depression (β = −0.03; SE, 0.01; *P* = .006).

**Conclusions and Relevance:**

In this study, younger adolescents, those with parents with lower education, or those with low genetic liability for depression appeared more vulnerable when being quarantined several times. These findings emphasize the need for targeted support strategies to better protect adolescent well-being during future crises. Adolescents who experienced increased mental distress during the COVID-19 pandemic may be at risk of continued mental health problems and in need of ongoing support.

## Key Points

**Question:**

How were stringent public health measures and quarantine during the COVID-19 pandemic associated with mental distress among Norwegian adolescents, and did vulnerability factors moderate the associations?

**Findings:**

In this cohort study of 7787 participants, stricter public health measures and quarantine were associated with adolescent mental distress. Younger adolescents, those with parents with lower education, or those with lower genetic liability to depression showed particularly elevated mental distress by more frequent quarantines.

**Meaning:**

These findings suggest the need for targeted support to protect adolescent well-being during future crises and emphasize the possible ongoing risk of mental health problems in adolescents following the COVID-19 pandemic.

## Teaser

This cohort study examines the association of mental health and the public health measures and quarantine experiences during the COVID-19 pandemic among Norwegian adolescents.

## Introduction

Adolescence represents a transitional period, in which some individuals experience the emergence of mental health disorders, such as anxiety, depression, or eating disorders,^1^ with a typical age of onset between 12 to 25 years.^2^ For example, the period includes developmental changes in cognitive and socioemotional regulation mechanisms that influence decision-making, peer relationships, and well-being.^3,4^ On March 12, 2020, in response to the COVID-19 pandemic, the Norwegian government implemented several public health measures. These included school closures, stay-at-home mandates, and travel restrictions. Although these public measures reduced the spread of the COVID-19 virus, the cessation of after-school activities^5^ and isolation from friends^6^ could pose threats to adolescent mental health.

In addition to a general trend of increasing mental health problems among young people during the last decades,^7,8,9^ numerous studies^10,11,12,13,14^ have reported an increase in symptoms of anxiety and depression during the pandemic compared with prepandemic levels, as summarized in a recent meta-analysis based on 53 longitudinal studies.^15^ Girls tend to show worse mental health during the pandemic compared with boys,^16^ and research also points to factors, such as older age,^17,18^ lower parental education^19,20^ and preexisting mental health problems,^21^ as possible vulnerability factors.

Although studies have investigated the associations of restrictive public health measures^22^ and quarantine^23,24^ with mental health during the COVID-19 pandemic, there is a lack of evidence from adolescent samples. Moreover, except for studies^23,25^ suggesting that girls had a worse experience during quarantine than boys, few studies have investigated how individual-level characteristics are associated with mental health outcomes following the implementation of public health measures. Knowledge of potential adverse effects is crucial for policy makers to consider when introducing public health measures to limit spread of communicable diseases in the future. For instance, considering the varying risks and impacts across different people, quarantine measures might be adjusted so that they are not mandatory for vulnerable groups.

It is hard to disentangle the role of the COVID-19 pandemic from the increase in mental health problems among adolescents, which were already on the rise. However, while the pandemic impacted everyone, the stringency of restrictions varied over time. In this study, we used a national restriction stringency index, capturing daily data from April 2020 to February 2021, to investigate how changes in public health measures corresponded with adolescent mental distress. By capturing variance in restrictions, we provide insight into a possible direct mechanism of the pandemic. We further examined the association of recent quarantine and quarantine frequency with mental distress. Subsequently, we explored whether vulnerability factors, such as female sex, higher age, low parental education, prepandemic mental health problems, and genetic liability for mental health conditions, might moderate these associations.

## Methods

### Study Design and Sample

**Figure 1.**
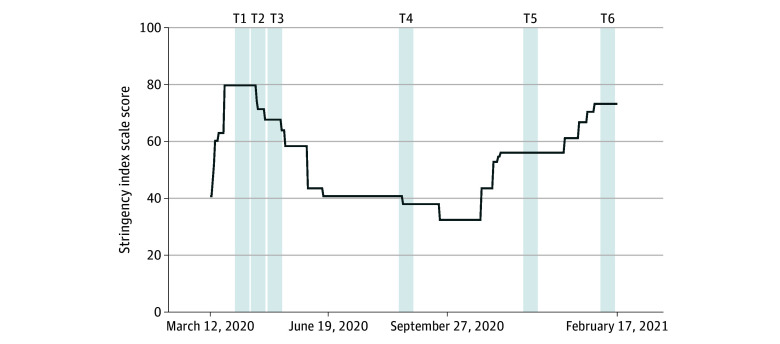
Measure of Mental Distress and COVID-19 Restriction Level in Norway Specific dates for the 6 time points: (1) March 31 to April 14, 2020; (2) April 14 to 29, 2020; (3) April 29 to May 12, 2020; (4) August 19 to September 1, 2020; (5) December 8 to December 21, 2020; (6) February 2 to Febuary 17, 2021. The gray bars indicate time points when adolescents responded to the Hopkin’s Symptom Checklist (SCL-5) measure of mental distress. The line indicates the stringency index scaled from 0 to 100 (100 being the strictest), extracted from Hale et al.^34^

Between March 2020 and February 2021, participants aged 16 to 18 years enrolled in the Norwegian Mother, Father, and Child Cohort Study (MoBa) were invited to complete biweekly COVID-19 surveys. Six of these included a measure of mental distress (Figure 1). MoBa is a population-based pregnancy cohort study conducted by the Norwegian Institute of Public Health.^26,27^ Participants were recruited from all over Norway from 1999 to 2008. The women consented to participation in 41% of the pregnancies. The cohort includes approximately 114 500 children, 95 200 mothers, and 75 200 fathers. Blood samples were collected from the umbilical cord during delivery.^28^ Genotype data were quality-controlled using the MoBa PsychGen pipeline.^29^ Linked data from the Norwegian Patient Registry provided diagnostic information from specialist health care from 2008 to 2020 using the *International Statistical Classification of Diseases and Related Health Problems, Tenth Revision *(*ICD-10*) codes.^30^ Data from the Payment of Health Reimbursements Database contain codes from the International Classification of Primary Care–2^31^ between 2006 to 2020. Information about parental education was attained from Statistics Norway.

The establishment and initial data collection of MoBa were based on a license from the Norwegian Data Protection Agency and approval from The Regional Committees for Medical and Health Research Ethics (REK). The MoBa cohort is currently regulated by the Norwegian Health Registry Act. The current study was approved by REK. This longitudinal cohort study followed the Strengthening the Reporting of Observational Studies in Epidemiology (STROBE) reporting guideline.^32^

Of 17 250 adolescents invited to the first COVID-19 survey, 51% (8663) responded. Restricting to adolescents responding to at least 1 questionnaire assessing mental distress, with data on covariates and genetic data, our analytic sample consisted of 7787 adolescents (eFigure in Supplement 1). Supplementary analyses were run on a subsample of 2390 individuals who also responded to a (prepandemic) questionnaire at age 14 years. The eMethods in Supplement 1 provides details on all included measures.

### Outcome

Mental distress was measured by the 5-item version of the Hopkin’s Symptom Checklist (SCL-5).^33^ The SCL-5 consists of 2 items tapping anxiety and 3 tapping depression symptoms.

### Exposure

The stringency of public health measure was extracted from the Oxford COVID-19 Government Response Tracker,^34^ which combines information from 9 metrics, including closures of schools, workplaces and public transport, cancellation of public events, and international travel controls. The index was scored from 0 to 100, with higher score indicating stricter measures. Scores were matched to each SCL-5 report according to the response date.

From March 2020, the Norwegian government mandated a 14-day quarantine for those returning from travel abroad or who had been in contact with confirmed COVID-19 cases. The first couple of weeks with lockdown did not include mandated quarantine, just a recommendation to stay at home. During quarantine, individuals had to stay at home but going outside and necessary shopping (with social distancing) was allowed. Individuals with symptoms or a positive COVID-19 test had to isolate at home but could complete the remaining time in quarantine following a negative test. We created 2 variables based on the question: “Have you been quarantined/in isolation during the last 14 days?” First, recent quarantine (yes or no) was registered according to being in quarantine during the last 2 weeks. Second, frequency of quarantine was calculated as the number of times quarantined (0 to 5 or more) at the time of data collection. Details regarding quarantine rules or guidelines in Norway can be found in the eMethods in Supplement 1.

Time (ie, days since March 12, 2020) was added as a continuous covariate. Sex (ie, male or female) and age (ie, age 16, 17, and 18 years) at each measurement were included as categorical variables. Prepandemic anxiety and depression (yes or no) were defined based on diagnostic codes in primary and specialist health care (eMethods in Supplement 1). Parental education was included as a continuous variable.

Polygenic scores (PGS) indicating genetic liability for anxiety, depression, anorexia nervosa, and neuroticism were generated. Measures of prepandemic mental distress and eating problems (available for a subsample) were included as covariates in separate supplementary analyses.

### Statistical Analyses

We ran 11 linear mixed-effect models (M0-M10) (eTable 1 in Supplement 1) using the lme4 package^35^ in R version 4.1.2 (R Project for Statistical Computing)^36^ with RStudio.^37^ We included participants with between 1 and 6 responses to SCL-5 in multilevel models using restricted information maximum likelihood estimation. All models have a hierarchical structure, estimating parameters at 2 levels, allowing us to investigate variance between (level 2) and within individuals (level 1). On level 1, we include variables that can vary across observations (eg, time, stringency). On level 2, we include variables related to the individual (eg, sex, prepandemic anxiety or depression). Nested models were compared pairwise using the analysis of variance function (anova) from the R stats package.^36^ We validated the model fit by comparing variance explained using marginal and conditional pseudo-R-squared values.^38^

The baseline model (M0) included a random intercept of the participants’ identification number to specify that there were repeated SCL-5 measures for each participant. We ran 2 nested models to estimate the association between public health measures and mental distress. In the first model (M1), we included stringency index and time at level 1 (ie, as fixed effects). We compared the fit of M1 with M0 to assess whether stringency and time were associated with mental distress (ie, testing whether additional terms in M1 explained a significant proportion of the residual variance from M0). In the second model (M2), we also included stringency index at level 2 (ie, as a random slope). This allowed participants to have different linear effects associated with the stringency index accounting for potential unexplained between-individual variance. We then compared the fit of M2 with M1 to assess whether the trajectories differed sufficiently in response to changes in the stringency index. From the best-fitting model (M1 vs M2) we used the significance (*α* = .05) of the estimated fixed effect of the stringency index as the basis of the inference for our first hypothesis.

To investigate to what extent the association between stringency level and mental distress differs according to selected characteristics, each moderator (sex, age, parental education, prepandemic anxiety or depression, and 4 PGS) was added as level 2 fixed effects to our best-fitting model (M1 or M2) in the same model (M3). Next, in M4, we added cross-level interaction terms between each moderator and the stringency index. The model fit of M4 was compared with M3. If M4 was the best fitting, we interpreted individual interaction terms.

To estimate the association between recent quarantine and mental distress, we added recent quarantine as level 1 fixed effects in the best-fitting model (M1 or M2) in M5. To investigate moderating effects, covariates were added in M6 and interaction terms between moderators and recent quarantine in M7. Frequency of quarantine was modeled similarly to recent quarantine, adding level 1 fixed effects in M8, covariates in M9, and interaction terms in M10.

For supplementary analyses, we included 2 additional possible moderators reported by a subsample at age 14 years. Prepandemic mental distress and eating problems into our previously specified models. In supplementary (S) models, we added self-reported prepandemic measures as fixed effects and interaction terms with stringency (M-S1-S2), recent quarantine (M-S3-S4), and frequent quarantine (M-S5-S6).

All nested models were run using the same sample. We compared descriptive characteristics across samples: (1) all MoBa participants aged 16 to 18 years, (2) sample in M1 to M4, (3) sample in M5 to M10, and (4) sample in S1 to S6.

To correct for multiple testing across 2 different quarantine measures we used Bonferroni correction to adjust the α level (*α* = .025, found by .05/2). We used maximum likelihood estimation for analysis of variance comparisons. Data were analyzed from October 2022 to October 2023. Statistical significance was set at *P* < .05, and all tests were 2-sided.

## Results

This study includes 7787 adolescents (4473 female [57%]; mean [SD] age, 17.0 [0.6] years). Most participants (5342 [69%]) were 17 years old, 3987 (51%) responded to SCL-5 at least 3 times, and 1730 (22%) had experienced quarantine. The mean (SD) SCL-5 score was 1.53 (0.56) with 1242 (16%) scoring above the established cut-off of 2.0.^39^ The mean (SD) stringency level during the first pandemic year was 51.8 (14.3), with the highest level at 79.6 in April 2020 and the lowest level at 32.4 from September to October 2020 (Figure 1). Descriptive characteristics are presented in Table 1.

**Table 1.** Sample Selection and Descriptive Characteristics^a^

Variables	Individuals, No. (%)			
	All 16-18-y-olds	Models 0-4	Models 5-10	Supplementary models
Sample	20 432	7787	7780	2390
Observations	NA	20 302	20 251	7610
Sex				
Male	10 393 (50.9)	3314 (42.6)	3310 (42.5)	960 (40.2)
Female	10 039 (49.1)	4473 (57.4)	4470 (57.5)	1430 (59.8)
Age, y				
16	3433 (16.8)	1371 (17.6)	1370 (17.6)	615 (25.7)
17	13 286 (65.0)	5342 (68.6)	5338 (68.6)	1480 (61.9)
18	3713 (18.2)	1074 (13.8)	1072 (13.8)	295 (12.3)
Prepandemic anxiety or depression				
No	18 459 (90.3)	7114 (91.4)	7107 (91.3)	2221 (92.9)
Yes	1973 (9.7)	673 (8.6)	673 (8.7)	169 (7.1)
Prepandemic anxiety				
No	19 092 (93.4)	7320 (94.0)	7313 (94.0)	2279 (95.4)
Yes	1340 (6.6)	467 (6.0)	467 (6.0)	111 (4.6)
Prepandemic depression				
No	19 463 (95.3)	7466 (95.9)	7459 (95.9)	2299 (96.2)
Yes	969 (4.7)	321 (4.1)	321 (4.1)	91 (3.8)
Mother’s education				
Compulsory or none	1468 (7.2)	337 (4.3)	337 (4.3)	47 (2.0)
Upper secondary	6250 (30.7)	2107 (27.1)	2102 (27.0)	535 (22.4)
Bachelor’s	9844 (48.3)	4054 (52.1)	4053 (52.1)	1332 (55.8)
Master’s or PhD	2822 (13.8)	1283 (16.5)	1282 (16.5)	474 (19.8)
Missing	51 (0.2)	6 (0.1)	6 (0.1)	2 (0.1)
Father’s education				
Compulsory or none	1431 (9.0)	449 (7.0)	448 (7.0)	117 (5.8)
Upper secondary	7378 (46.6)	2802 (43.7)	2799 (43.7)	798 (39.4)
Bachelor’s	4626 (29.2)	2025 (31.6)	2023 (31.6)	691 (34.1)
Master’s or PhD	2400 (15.2)	1138 (17.7)	1138 (17.6)	421 (20.8)
Missing	4597 (22.5)	1373 (17.6)	1372 (17.6)	363 (15.2)
Mean (SD) SCL-5^b^	NA	1.53 (0.56)	1.53 (0.56)	1.52 (0.56)
SCL-5 cut off (2.0)				
No	NA	6545 (84.1)	6538 (84.0)	2008 (84.0)
Yes	NA	1242 (15.9)	1242 (16.0)	328 (16.0)

Abbreviations: *ICD-10*, *International Statistical Classification of Diseases and Related Health Problems, Tenth Revision*; ICPC-2, International Classification of Primary Care; SCL-5, Hopkins symptom checklist (5 item version).

^a^ Supplementary model includes self-reported prepandemic measures. All 16- to 18-year-olds in Norwegian Mother, Father, and Child Cohort Study were invited to answer these questionnaires. Prepandemic anxiety includes 1 of the following diagnostic codes from the *ICD-10* (ie, F40, F41, or F93) or the ICPC-2 (ie, P74 or P79). Prepandemic depression includes 1 of the following diagnostic codes: *ICD-10* (ie, F32, F33, F34, or F92) or *ICPC-2* (ie, P76).

^b^ Mean score based on a SCL-5 scale from 1 to 4.

### Public Health Measures

**Figure 2.**
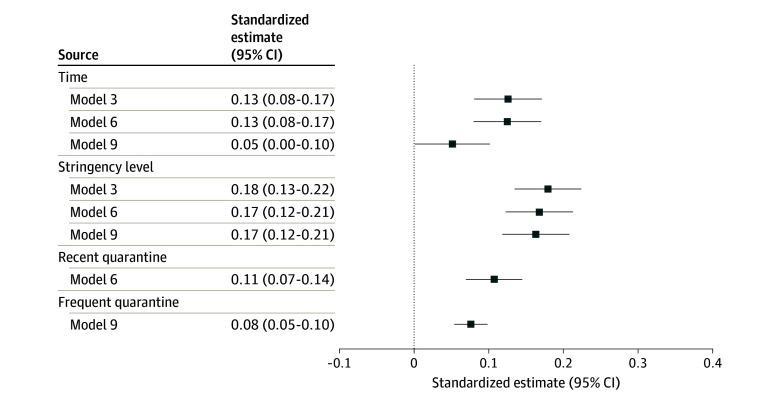
Multilevel Model Main Effect Estimates for Time, Stringency Level, and Quarantine Standardized model estimates from 3 separate models. Standardized effects data can be found in eTable 2 in Supplement 1 (model 3) and Table 3 (models 3 and 6).

Including the stringency index as a fixed effect (M1) provided a better fit to our data compared with the baseline model (M0). M2, with a random slope for stringency, explained additional 3.2% of the variance. Including covariates (M3) further improved the fit to our data. Based on M3, higher stringency (β = 0.18; SE, 0.02; *P* < .001) and time (β = 0.13; SE, 0.02; *P* < .001) were associated with increased mental distress (Figure 2). All M3 parameter estimates can be found in eTable 2 in Supplement 1. Adding interaction terms (M4) did not improve the fit to our data (eTable 3 in Supplement 1). For supplementary models including self-reported prepandemic measures from a smaller sample, the model without interaction terms was best fitting (eTable 4 and 5 in Supplement 1).

### Recent Quarantine

Including covariates (M6) improved the fit of the model estimating associations between recent quarantine and mental distress (M5) but adding interaction terms (M7) did not. In the best-fitting model (M6) adolescents who had been quarantined reported more mental distress (β = 0.11; SE, 0.02; *P* < .001) (Table 2). Parameter estimates are shown in Table 3. For supplementary models including prepandemic measures, the model without interaction terms was the best fitting (eTable 6 to 7 in Supplement 1).

**Table 2.** Model Fit Comparison of Models 5 to Model 10^a^

Model	Name	AIC	BIC	χ^2^ Difference	Degrees of freedom	*P* value	Comparison model
5	Fixed effect for recent quarantine	47 519	47 583	NA	NA	NA	NA
6	Covariates	46 460	46 587	1075.4	8	<.001	5
7	Interactions	46 470	46 660	6.1	8	.63	6
8	Fixed effect for quarantine amount	47 501	47 564	NA	NA	NA	NA
9	Covariates	46 446	46 573	1070.2	8	<.001	8
10	Interactions	46 437	46 627	25.3	8	.001	9

Abbreviations: AIC, Akaike information criterion; BIC, bayesian information criterion; NA, not applicable.

^a^ Model comparison using analysis of variance. The variable time was added to each model.

**Table 3.** Main Outcomes and Interactions of Quarantine on Mental Distress

Variable	Model 5		Model 6		Model 7		Model 8		Model 9		Model 10	
	β (SE)	*P* value	β (SE)	*P* value	β (SE)	*P* value	β (SE)	*P* value	β (SE)	*P* value	β (SE)	*P* value
Main exposure associations												
Recent quarantine	0.104 (0.019)	<.001	0.107 (0.019)	<.001	0.209 (0.069)	.003	NA	NA	NA	NA	NA	NA
Time	0.192 (0.019)	<.001	0.125 (0.023)	<.001	0.124 (0.023)	<.001	NA	NA	NA	NA	NA	NA
Interactions												
Recent quarantine × sex	NA	NA	NA	NA	−0.064 (0.040)	.11	NA	NA	NA	NA	NA	NA
Recent quarantine × age	NA	NA	NA	NA	−0.014 (0.033)	.67	NA	NA	NA	NA	NA	NA
Recent quarantine × prepandemic anxiety or depression^a^	NA	NA	NA	NA	−0.059 (0.067)	.38	NA	NA	NA	NA	NA	NA
Recent quarantine × parental education	NA	NA	NA	NA	−0.020 (0.026)	.43	NA	NA	NA	NA	NA	NA
AN PGS	NA	NA	NA	NA	0.017 (0.019)	.37	NA	NA	NA	NA	NA	NA
Recent quarantine × anxiety PGS	NA	NA	NA	NA	−0.019 (0.020)	.33	NA	NA	NA	NA	NA	NA
Recent quarantine × depression PGS	NA	NA	NA	NA	0.014 (0.020)	.48	NA	NA	NA	NA	NA	NA
Recent quarantine × neuroticism PGS	NA	NA	NA	NA	0.011 (0.019)	.58	NA	NA	NA	NA	NA	NA
Main exposure associations												
Quarantine frequency	NA	NA	NA	NA	NA	NA	0.080 (0.012)	<.001	0.076 (0.011)	<.001	0.190 (0.038)	<.001
Time	NA	NA	NA	NA	NA	NA	0.114 (0.022)	<.001	0.051 (0.026)	.04	0.055 (0.026)	.03
Interactions												
Quarantine frequency × sex	NA	NA	NA	NA	NA	NA	NA	NA	NA	NA	0.006 (0.023)	.79
Quarantine frequency × age	NA	NA	NA	NA	NA	NA	NA	NA	NA	NA	−0.037 (0.014)	.008
Quarantine frequency × prepandemic anxiety or depression^a^	NA	NA	NA	NA	NA	NA	NA	NA	NA	NA	−0.032 (0.032)	.31
Quarantine frequency × parental education	NA	NA	NA	NA	NA	NA	NA	NA	NA	NA	−0.036 (0.014)	.007
Quarantine frequency × AN PGS	NA	NA	NA	NA	NA	NA	NA	NA	NA	NA	0.012 (0.010)	.24
Quarantine frequency × anxiety PGS	NA	NA	NA	NA	NA	NA	NA	NA	NA	NA	−0.008 (0.011)	.45
Quarantine frequency × MDD PGS	NA	NA	NA	NA	NA	NA	NA	NA	NA	NA	−0.027 (0.010)	.006
Quarantine frequency × neuroticism PGS	NA	NA	NA	NA	NA	NA	NA	NA	NA	NA	0.008 (0.010)	.45
Random effects												
ICC	0.723		0.689		0.689		0.772		0.689	NA	0.689	NA
Marginal *R^2^* (conditional *R^2^*)	0.003 (0.724)		0.108 (0.723)		0.108 (0.723)		0.005 (0.723)		0.109 (0.723)	NA	0.110 (0.723)	NA

Abbreviations: AN, anorexia nervosa; ICC, interclass correlation coefficient; MDD, major depressive disorder; NA, not applicable; PGS, polygenic score.

^a^ Prepandemic anxiety or depression includes 1 of the following diagnostic codes from *International Statistical Classification of Diseases and Related Health Problems, Tenth Revision* (ie, F32, F33, F34, F40, F41, F93, or F94) or International Classification of Primary Care-2 (ie, P74, P76, or P79).

### Quarantine Frequency

Including covariates (M9) improved the fit of the model estimating associations between quarantine frequency and mental distress (M8). Being frequently quarantined was associated with mental distress (β = 0.08; SE, 0.01; *P* < .001). Adding interaction terms (M10), showed an improved model fit. Significant interactions were observed between frequency of quarantines and age (β = −0.04; SE, 0.01; *P* < .01), parental education (β = −0.04; SE, 0.01; *P* < .01) and PGS for depression (β = −0.03; SE, 0.01; *P* < .01) on mental distress (Table 2). Parameter estimates are shown in Table 3. For supplementary models, the model with interaction terms (M-S6) provided the best fit (eTable 6 to 7 in Supplement 1). Adolescents with higher prepandemic mental distress had a steeper increase in mental distress when experiencing more frequent quarantines (β = 0.03; SE, 0.02; *P* < .05), but this did not remain significant after correcting for multiple testing.

## Discussion

In this longitudinal cohort study, stricter public health measures during the COVID-19 pandemic were associated with adolescent mental distress. Contrary to expected, this association was not moderated by sex, age, prepandemic anxiety or depression, parental education, or genetic liability for mental health conditions. Adolescents who had recently experienced quarantine reported more mental distress, and there was a dose-response association between the number of times quarantined and mental distress. This association was more pronounced among 16-year-olds, those with parents with lower education, and adolescents with a lower depression PGS. Our findings aligned with studies that showed increased mental distress among adolescents during times of stricter measures.^10,11,12^ A meta-analysis^22^ found a linear association between the stringency of measures and depression or anxiety symptoms. However, this review primarily focused on adult participants and included only a few smaller adolescent samples (ie, less than 300 adolescents).

Aligning with previous research,^23,24^ we found that both recent and frequent quarantine were associated with mental distress. Association of time was halved when frequency of quarantine was added to the model, indicating that much of the association with time was driven by participants being quarantined. Elevated mental distress during strict public health measures and quarantine could be due to several mechanisms. Social distancing measures can disrupt social connectedness with friends and grandparents, relationships shown to be important for adolescent well-being.^40^ Reduced in-person social interactions and more time spent indoors may increase feelings of loneliness and impact mental health.^6^ The public health measures can also lead to uncertainty, financial hardships, break-up of daily routines, and changes in dietary and sleep patterns, which are factors associated with adolescent mental distress.^23,41^ Physical activity is associated with mental health^42^ and the lack of organized sports might also have contributed to the association. Strict public health measures and quarantine often followed times of rising COVID-19 cases. Therefore, there is a possibly that the associations reported in our study reflects fears associated with this (eg, of infection or death of self or loved ones).

Our sample reported higher levels of mental distress than in a similar Norwegian sample in 1998, which reported a score of 1.38 on a scale of 1 to 4.^39^ This difference could be attributed to the general increasing trend of mental health problems or heightened levels during the COVID-19 pandemic.^7,8,9^ We expected mental distress to increase across adolescent development,^7,8,9^ which is in line with the observed main effect of age. The association between frequency of quarantine and mental distress was stronger among younger adolescents compared with older adolescents. In Norway, adolescents aged 16 years are typically in their first year of high school and may be more vulnerable to social isolation, given that their peer networks are likely less established.

Adolescents with parents with lower education showed a steeper increase in mental distress with frequent quarantine, highlighting the possible role of socioeconomic disparity on adolescents’ well-being. Several factors could contribute to this, such as parental income loss or more confined living arrangements, possibly intensifying stress among adolescents when quarantined.

As expected, genetic liability to mental disorders was positively associated with mental distress, but only for the depression PGS. Regarding interaction effects, we found that adolescents with lower depression PGS showed a steeper increase in mental distress with frequent quarantine. Social isolation might be linked to more distress in those not generally distressed, perhaps due to different coping skills and experiences. Additionally, the social network of those with low depression liability might have been stronger and more affected by the COVID-19 pandemic. Adolescents with high PGS may have struggled more before the pandemic (eg, with low social contact, or by being bullied), and some may have experienced a relief by having fewer social or leisure time obligations.^43^ Our findings aligned with studies showing that adolescents with prepandemic psychiatric symptoms showed an decrease in symptoms^43^ and that children with preexisting mental health disorders were less in contact with health care services during the pandemic.^44^

### Limitations

This study has limitations. First, MoBa has a 41% initial response rate and predominantly consists of well-educated, healthy families.^45^ Approximately 50% of their children participated in the COVID-19 data collections, and it is not known how well they represent the general adolescent population in Norway. Second, participation dropped from 51% to 17% throughout the 6 data collections, potentially causing selective attrition. However, comparing characteristics of our analytic samples to all adolescents invited did not indicate important differences. Third, observational data limits causal inference. However, the COVID-19 pandemic settings allowed for a natural experiment. The varying intensity of public health measures across time introduced exogenous variation. Fourth, some adolescents might have received mental health services during the pandemic possibly relieving mental distress. Fifth, while the β coefficients are generally small, it is important to consider the broader context in which these associations occur. In the global population, even small shifts in the normal distribution can translate into significant public health implications.^46^ Sixth, some participants may have interpreted the question about quarantine and isolation as also including voluntary isolation. Adolescents choosing to isolate voluntarily might also score higher on the distress scale. However, we do not believe this to be a major issue, both due to how these governmental measures were communicated in Norway, and due to the low frequency of quarantine and isolation in our sample. Seventh, only 22% reported ever being quarantined possibly limiting the generalizability to countries where quarantine was more prevalent. Future research should investigate regional and country differences in public health measures.

## Conclusion

These findings suggest that public health measures and quarantine experiences were associated with adolescent mental distress. In general, these associations were not moderated by vulnerability factors, except for the association with the frequency of quarantine. Younger adolescents, those with parents with lower education, and those with lower genetic risk for depression showed more mental distress with repeated quarantines. Insight into how public health measures are associated with adolescent mental health during the COVID-19 pandemic is critical to advance our knowledge and inform policy decisions in preparation for future global public health crises.

## Notes

Supplement 1. **eFigure. **Flow Chart of Study Participants
eMethods.
**eTable 1.** Model Equations for M0 to M10 **eTable 2.** Multilevel Model Estimates From Models 1 to 3 **eTable 3.** Model Fit Comparison—Stringency Index and Mental Distress **eTable 4.** Model Fit Comparison—Stringency Index and Mental Distress Including Self-Reported Prepandemic Measures **eTable 5.** Multilevel Model Estimates From Model S1 **eTable 6.** Model Fit Comparison—Including Self-Reported Prepandemic Measures **eTable 7.** Main Effects and Interaction Effects of Quarantine on Mental Distress Including Self-Reported Prepandemic Measures in Supplementary Analyses
eReferences.

Supplement 2.
Data Sharing Statement
