# Supplementary material for: COVID-19 Pandemic Quarantines and Mental Health Among Adolescents in Norway
Source: JAMA Netw Open. 2024 Jul 12;7(7):e2422189. doi: 10.1001/jamanetworkopen.2024.22189 (PMC11245726; doi:10.1001/jamanetworkopen.2024.22189)
Supplement: Supplement 1. — eFigure. Flow Chart of Study Participants eMethods. eTable 1. Model Equations for M0 to M10 eTable 2. Multilevel Model Estimates From Models 1 to 3 eTable 3. Model Fit Comparison—Stringency Index and Mental Distress eTable 4. Model Fit Comparison—Stringency Index and Mental Distress Including Self-Reported Prepandemic Measures eTable 5. Multilevel Model Estimates From Model S1 eTable 6. Model Fit Comparison—Including Self-Reported Prepandemic Measures eTable 7. Main Effects and Interaction Effects of Quarantine on Mental Distress Including Self-Reported Prepandemic Measures in Supplementary Analyses eReferences. [file jamanetwopen-e2422189-s001.pdf]

Supplemental Online Content

Pettersen JH, Hannigan LJ, Gustavson K, et al. COVID-19 pandemic quarantines and mental health among adolescents in Norway. *JAMA Netw Open*. 2024;7(7):e2422189. doi:10.1001/jamanetworkopen.2024.22189

eFigure 1. Flow Chart of Study Participants

eMethods.

eTable 1. Model Equations for M0 to M10

eTable 2. Multilevel Model Estimates from Models 1 to 3

eTable 3. Model Fit Comparison—Stringency Index and Mental Distress

eTable 4. Model Fit Comparison—Stringency Index and Mental Distress Including Self-Reported Prepandemic Measures

eTable 5. Multilevel Model Estimates From Model S1

eTable 6. Model Fit Comparison—Including Self-Reported Prepandemic Measures

eTable 7. Main Exposure-Associations and Interactions of Quarantine on Mental Distress Including Self-Reported Prepandemic Measures in Supplementary Analyses

eReferences

This supplemental material has been provided by the authors to give readers additional information about their work.

© 2024 Pettersen JH et al. *JAMA Network Open.*

eFigure 1. Flow chart of study participants

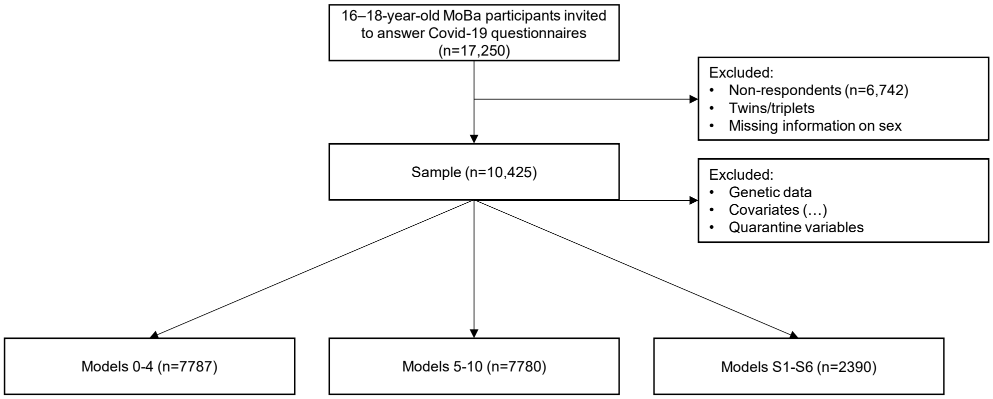

eMethods. Further Method Details

Outcome measure SCL-5 (mental distress)

The SCL-5 originates from the 25-item SCL, validated in several populations[1,2], shown to be highly correlated (r = 0.92) with SCL-5. The SCL-5 has an alpha reliability of 0.87. Each item is scored on a scale of 1 (not bothered) to 4 (very bothered), referring to experiences during the last two weeks[3]. For each participant at each of the six time points, a mean score was first calculated and then multiplied with the total number of items, with a higher score indicating a higher level of mental distress. The SCL-5 score was then log-transformed and standardized. If respondents answered fewer than two items, their score was not computed, and the data was considered missing.

Stringency index

The Oxford COVID-19 Government Response Tracker (OxCGRT) index consists of nine metrics: school closures, workplace closures, cancellation of public events, restrictions on public gatherings, public transport closures, public information campaigns, restrictions on internal movements, and international travel controls.

Quarantine

During quarantine, individuals had to stay at home and not attend school or work physically or travel with public transportation. Going outside, necessary shopping (with 1-meter social distancing) was allowed, and individuals living in the same household were not quarantined. Quarantine length was reduced to 10 days in May 2020, and 7 days in December 2020 (if you could provide two negative Covid-19 tests)[4].

Parental education

A variable was created for parental education based on data from Statistics Norway (SSB). We combined mothers' and fathers' education level by keeping the highest parental education level for each child. The variable was scored from 0-3 (0 = none or compulsory, 1 = upper secondary, 2 = Bachelor's degree, 3 = Master's degree or Ph.D.).

NPR and KUHR diagnostic codes for pre-pandemic anxiety or depressive disorder

Using the "Phenotools" R package[5], individuals were coded with "Yes" on the variable pre-pandemic anxiety/depression if they ever were diagnosed in NPR with ICD-10 codes for depressive episode (F32), recurrent depressive episode (F33), persistent mood disorder (F34), phobic anxiety disorders (F40), other anxiety disorders (F41), mixed disorders of conduct or emotions (F92), or emotional disorders with onset specific to childhood (F93), or ever registered with the following ICPC-2 codes in KUHR: depressive disorder (P76), anxiety disorder/anxiety state (P74) or phobia/obsessive-compulsive disorder (P79).

SCL-5 and eating problems from a questionnaire answered by a subsample at age 14

© 2024 Pettersen JH et al. *JAMA Network Open.*

A variable was created for SCL-5 measured at 14 years following the same procedure as the outcome measure. Additionally, a variable was created measuring eating problems from the Eating Disorder Examination Questionnaire (EDE-Q).[6] The first five items of the EDE-Q were: *"over the past 4 weeks, how often 1) have you been deliberately trying to limit the amount of food you eat to influence your shape or weight? 2) have you tried to follow definite rules regarding what you can eat, in order to influence your shape or weight? 3) have you had a definite fear of losing control over eating? 4) has thinking about food, eating or calories made it very difficult to concentrate on things you are interested in? 5) have you eaten secretly?"* The last three items were: *1) how dissatisfied have you been with your shape? 2) how uncomfortable have you felt seeing your own body? 3) how uncomfortable have you felt about others seeing your shape or figure?* All responses were scored from 0-3 (ranging from "Not at all"/"Never" to "Much"/"Often"). Mean scores were calculated for those who completed at least 4 items. The scores were then log-transformed and standardized.

Polygenic scores

Polygenic scores (PGS) were created by calculating the sum of an individual's risk alleles, weighted by the risk alleles effect size estimated from a genome-wide association study (GWAS) on that specific trait.[7] PGS were then created for anxiety,[8] depression,[9] anorexia nervosa (AN),[10] and neuroticism,[11] using PRSice2.[12] The summary statistics for each PGS were filtered for minor allele frequencies (MAF>1%) and INFO threshold >.8. A range of p-value thresholds was then selected to create the PRS (<5e-08, <5e-07, <5e-06, <5e-05, <0.0005, <0.001, <0.005, <0.01, <0.1, <.05, and 1). Then, we used principal component analysis (PCA) on these PGS (calculated based on different thresholds) with the first PGS principal component as the predictor variable[13]. This approach reduces the risk of type 1 error and overfitting. The PGS are continuous and normally distributed variables.

© 2024 Pettersen JH et al. *JAMA Network Open.*

eTable 1. Model Equations for M0-M10

Model	Level 1 equation	Level 2 equation	Combined equation	Description
**0**	$\gamma_{ij} = \beta_{0j} + e_{ij}$	$\beta_{0j} = \gamma_{00} + u_{0j}$	$\gamma_{ij} = \gamma_{00} + u_{0j} + e_{ij}$	Baseline model with random intercept of ID
**1**	$\gamma_{ij} = \beta_{0j}$ $+\beta_{1j}(Stringency)$ $+\beta_{2j}(Time) + e_{ij}$	$\beta_{0j} = \gamma_{00} + u_{0j},$ $\beta_{1j} = \gamma_{10},$ $\beta_{2j} = \gamma_{20}$	$\gamma_{ij} = \gamma_{00} + \gamma_{10}(Stringency)$ $+ \gamma_{20}(Time) + u_{0j} + e_{ij}$	Added fixed effect of time and stringency index
**2**	$\gamma_{ij} = \beta_{0j}$ $+\beta_{1j}(Stringency)$ $+\beta_{2j}(Time) + e_{ij}$	$\beta_{0j} = \gamma_{00} + u_{0j},$ $\beta_{1j} = \gamma_{10} + u_{1j},$ $\beta_{2j} = \gamma_{20}$	$\gamma_{ij} = \gamma_{00}$ $+(\gamma_{10} + u_{10})(Stringency)$ $+\gamma_{20}(Time) + U_{0j} + e_{ij}$	Random slope for stringency index
**3**	$\gamma_{ij} = \beta_{0j}$ $+\beta_{1j}(Stringency)$ $+\beta_{2j}(Time)$ $+\beta_{3j}(\boldsymbol{Cov}) + \cdots + e_{ij}$	$\beta_{0j} = \gamma_{00} + u_{0j},$ $\beta_{1j} = \gamma_{10} + u_{1j},$ $\beta_{2j} = \gamma_{20},$ $\beta_{3j} = \gamma_{30}, \dots$	$\gamma_{ij} = \gamma_{00}$ $+(\gamma_{10} + u_{10})(Stringency)$ $+\gamma_{20}(Time) + \gamma_{30}(\boldsymbol{Cov}) + \cdots$ $+U_{0j} + e_{ij}$	Added level-1 covariates as fixed effects
**4**	$\gamma_{ij} = \beta_{0j}$ $+\beta_{1j}(Stringency)$ $+ \beta_{2j}(Time) + \beta_{3j}(\boldsymbol{Cov}) + \cdots$ $+ \beta_{4j}(Stringency$ $\times \boldsymbol{Cov}) + \cdots + e_{ij}$	$\beta_{0j} = \gamma_{00} + u_{0j},$ $\beta_{1j} = \gamma_{10} + u_{1j},$ $\beta_{2j} = \gamma_{20},$ $\beta_{3j} = \gamma_{30}, \dots,$ $\beta_{4j} = \gamma_{40}, \dots$	$\gamma_{ij} = \gamma_{00}$ $+(\gamma_{10} + u_{10})(Stringency)$ $+\gamma_{20}(Time) + \gamma_{30}(\boldsymbol{Cov}) + \cdots$ $+\gamma_{40}(Stringency \times \boldsymbol{Cov}) + \cdots$ $+U_{0j} + e_{ij}$	Added cross-level interaction terms
**5**	$\gamma_{ij} = \beta_{0j}$ $+\beta_{1j}(Stringency)$ $+ \beta_{2j}(Time)$ $+ \beta_{3j}(Recent\ quarantine)$ $+ e_{ij}$	$\beta_{0j} = \gamma_{00} + u_{0j},$ $\beta_{1j} = \gamma_{10} + u_{1j},$ $\beta_{2j} = \gamma_{20},$ $\beta_{3j} = \gamma_{30}$	$\gamma_{ij} = \gamma_{00}$ $+(\gamma_{10} + u_{10})(Stringency)$ $+\gamma_{20}(Time)$ $+ \gamma_{30}(Recent\ quarantine)$ $+U_{0j} + e_{ij}$	Added recent quarantine as level 1 fixed effect
**6**	$\gamma_{ij} = \beta_{0j}$ $+\beta_{1j}(Stringency)$ $+ \beta_{2j}(Time)$ $+ \beta_{3j}(Recent\ quarantine)$ $+ \beta_{4j}(\boldsymbol{Cov}) + \cdots + e_{ij}$	$\beta_{0j} = \gamma_{00} + u_{0j},$ $\beta_{1j} = \gamma_{10} + u_{1j},$ $\beta_{2j} = \gamma_{20},$ $\beta_{3j} = \gamma_{30},$ $\beta_{4j} = \gamma_{40}, \dots$	$\gamma_{ij} = \gamma_{00}$ $+(\gamma_{10} + u_{10})(Stringency)$ $+\gamma_{20}(Time)$ $+ \gamma_{30}(Recent\ quarantine)$ $+\gamma_{40}(\boldsymbol{Cov}) + \cdots + U_{0j} + e_{ij}$	With recent quarantine and covariates as level-1 fixed effects
**7**	$\gamma_{ij} = \beta_{0j}$ $+\beta_{1j}(Stringency)$ $+ \beta_{2j}(Time)$ $+ \beta_{3j}(Recent\ quarantine)$ $+ \beta_{4j}(\boldsymbol{Cov}) + \cdots$ $+ \beta_{5j}(Recent\ quarantine$ $\times \boldsymbol{Cov}) + \cdots + e_{ij}$	$\beta_{0j} = \gamma_{00} + u_{0j},$ $\beta_{1j} = \gamma_{10} + u_{1j},$ $\beta_{2j} = \gamma_{20},$ $\beta_{3j} = \gamma_{20},$ $\beta_{4j} = \gamma_{40}, \dots,$ $\beta_{5j} = \gamma_{50}, \dots$	$\gamma_{ij} = \gamma_{00}$ $+(\gamma_{10} + u_{10})(Stringency)$ $+\gamma_{20}(Time)$ $+ \gamma_{30}(Recent\ quarantine)$ $+\gamma_{40}(\boldsymbol{Cov}) + \cdots$ $+\gamma_{50}(Recent\ quarantine$ $\times \boldsymbol{Cov})$ $+ \cdots + U_{0j} + e_{ij}$	Added interaction terms with recent quarantine

© 2024 Pettersen JH et al. *JAMA Network Open.*

eTable 1. Model equations for M0-M10

**8**	$\gamma_{ij} = \beta_{0j}$ $+\beta_{1j}(Stringency)$ $+\beta_{2j}(Time)$ $+\beta_{3j}(Frequent\ quarantine)$ $+e_{ij}$	$\beta_{0j} = \gamma_{00} + u_{0j}$, $\beta_{1j} = \gamma_{10} + u_{1j}$, $\beta_{2j} = \gamma_{20}$, $\beta_{3j} = \gamma_{30}$	$\gamma_{ij} = \gamma_{00}$ $+(\gamma_{10} + u_{10})(Stringency)$ $+\gamma_{20}(Time)$ $+\gamma_{30}(Frequent\ quarantine)$ $+U_{0j} + e_{ij}$	Added frequency of quarantine as level 1 fixed effect
**9**	$\gamma_{ij} = \beta_{0j}$ $+\beta_{1j}(Stringency)$ $+\beta_{2j}(Time)$ $+\beta_{3j}(Frequent\ quarantine)$ $+\beta_{4j}(\boldsymbol{Cov}) + \cdots + e_{ij}$	$\beta_{0j} = \gamma_{00} + u_{0j}$, $\beta_{1j} = \gamma_{10} + u_{1j}$, $\beta_{2j} = \gamma_{20}$, $\beta_{3j} = \gamma_{30}$, $\beta_{4j} = \gamma_{40}, \ldots$	$\gamma_{ij} = \gamma_{00}$ $+(\gamma_{10} + u_{10})(Stringency)$ $+\gamma_{20}(Time)$ $+\gamma_{30}(Frequent\ quarantine)$ $+\gamma_{40}(\boldsymbol{Cov}) + \cdots$ $+U_{0j} + e_{ij}$	With frequency of quarantine and covariates as level-1 fixed effects
**10**	$\gamma_{ij} = \beta_{0j}$ $+\beta_{1j}(Stringency)$ $+\beta_{2j}(Time)$ $+\beta_{3j}(Frequent\ quarantine)$ $+\beta_{4j}(\boldsymbol{Cov}) + \cdots$ $+\beta_{5j}(Frequent\ quarantine$ $\times \boldsymbol{Cov}) + \cdots + e_{ij}$	$\beta_{0j} = \gamma_{00} + u_{0j}$, $\beta_{1j} = \gamma_{10} + u_{1j}$, $\beta_{2j} = \gamma_{20}$, $\beta_{3j} = \gamma_{20}$, $\beta_{4j} = \gamma_{40}, \ldots,$ $\beta_{5j} = \gamma_{50}, \ldots$	$\gamma_{ij} = \gamma_{00}$ $+(\gamma_{10} + u_{10})(Stringency)$ $+\gamma_{20}(Time)$ $+\gamma_{30}(Frequent\ quarantine)$ $+\gamma_{40}(\boldsymbol{Cov}) + \cdots$ $+\gamma_{50}(Frequent\ quarantine$ $\times \boldsymbol{Cov})$ $+ \cdots + U_{0j} + e_{ij}$	Added interaction terms with frequency of quarantine

© 2024 Pettersen JH et al. *JAMA Network Open.*

eTable 2. Multilevel Model Estimates from Models 1-3

	Model 1		Model 2		Model 3	
	Std. Estimate (SE)	P value	Std. Estimate (SE)	P value	Std. Estimate (SE)	P value
**Main exposure associations**						
Restrictions	0.203 (0.019)	**<0.001**	0.206 (0.022)	**<0.001**	0.179 (0.023)	**<0.001**
Time	0.193 (0.020)	**<0.001**	0.193 (0.019)	**<0.001**	0.126 (0.023)	**<0.001**
Sex					0.575 (0.020)	**<0.001**
Age					0.069 (0.016)	**<0.001**
Pre-Pandemic anxiety/depression					0.420 (0.035)	**<0.001**
Parental education					0.008 (0.013)	0.54
AN PGS					-0.001 (0.010)	0.88
Anxiety PGS					0.019 (0.010)	0.06
Depression PGS					0.049 (0.010)	**<0.001**
Neuroticism PGS					0.042 (0.010)	**<0.001**
**Random effects**						
ICC		0.691		0.723		0.690
Marginal R^2/ Conditional R^2		0.002/ 0.692		0.003/ 0.724		0.107/ 0.723

Model 1 shows a model with level 1 fixed effects added. Model 2 included a random slope, and model 3 includes potential moderators. Model 4 (with interaction terms) is not included as it did not show improved fit to the data.

© 2024 Pettersen JH et al. *JAMA Network Open.*

eTable 3. Model Fit Comparison—Stringency Index and Mental Distress

Model	Name	AIC	BIC	Chi square difference	Δ Degrees of freedom	P value	Comparison model
**0**	Random intercept /null model	48011	48034				
**1**	Level 1 fixed effects of restriction level	47875	47915	139.3	2	**<0.001**	0
**2**	Random slope for restriction level	47641	47696	238.5	2	**<0.001**	1
**3**	Covariates	46582	46700	1075.1	8	**<0.001**	2
**4**	Interactions	46588	46770	10.1	8	0.26	3

Model fit comparison using ANOVA. Model 3 was significantly different from model 2 and seems to be the best fitting model. All models were adjusted for time.

© 2024 Pettersen JH et al. *JAMA Network Open.*

eTable 4. Model Fit Comparison—Stringency Index and Mental Distress Including Self-Reported Prepandemic Measures

Model	Name	AIC	BIC	Chi square difference	Δ Degrees of freedom	P value	Comparison model
**S1**	Covariates	16733	16851				
**S2**	Interactions	16744	16932	8.9	10	0.55	S1

Model comparison using ANOVA. Time was added to each model.

© 2024 Pettersen JH et al. *JAMA Network Open.*

eTable 5. Multilevel Model Estimates From Model S1

	Std. Estimate (SE)	P value
**Main exposure associations**		
Restrictions	0.118 (0.034)	**<0.001**
Time	0.097 (0.032)	**0.002**
Sex	0.324 (0.034)	**<0.001**
Age	0.100 (0.023)	**<0.001**
Pre-pandemic anxiety/depression	0.103 (0.063)	0.10
Pre-pandemic eating problems	0.114 (0.019)	**<0.001**
Pre-pandemic mental distress	0.345 (0.019)	**<0.001**
Parental education	0.012 (0.022)	0.60
AN PGS	-0.009 (0.016)	0.57
Anxiety PGS	-0.016 (0.016)	0.33
Depression PGS	0.054 (0.016)	**<0.001**
Neuroticism PGS	0.013 (0.016)	0.40
**Random effects**		
ICC	0.619	
Marginal R^2/Conditional R^2	0.252/0.715	

Model S1 includes potential moderators with pre-pandemic eating problems and pre-pandemic mental distress added as covariates.
Model S2 (with interaction terms) is not included as it did not show improved fit to the data.

© 2024 Pettersen JH et al. *JAMA Network Open.*

eTable 6. Model Fit Comparison—Including Self-Reported Prepandemic Measures

Model	Name	AIC	BIC	Chi square difference	Difference in degrees of freedom	P value	Comparison model
**S3**	Covariates	16728	16853				
**S4**	Interactions	16739	16933	8.7	10	0.56	S3
**S5**	Covariates	16730	16855				
**S6**	Interactions	16717	16911	33.4	10	**<0.001**	S5

Model comparison using ANOVA. Time was added to each model. Models S3-S4 and S5-S6 are compared to estimate moderating effect between recent quarantine and frequency of quarantine when self-reported pre-pandemic measures were added as covariates.

© 2024 Pettersen JH et al. *JAMA Network Open.*

eTable 7. Main Exposure-Associations and Interactions of Quarantine on Mental Distress Including Self-Reported Prepandemic Measures in Supplementary Analyses

	With covariates		With interactions	
	Std. Estimate (SE)	P value	Std. Estimate (SE)	P value
	Model S3		Model S4	
**Main exposure associations**				
Recent Quarantine	0.089 (0.032)	**0.006**	0.117 (0.125)	0.35
Time	0.097 (0.032)	**0.002**	0.097 (0.032)	**0.002**
**Interactions**				
Time*Recent Quarantine				
Time*Sex			-0.047 (0.074)	0.52
Time*Age			0.084 (0.051)	0.10
Time*Pre-pandemic anxiety/depression			-0.029 (0.128)	0.82
Time*Pre-pandemic eating problems			0.052 (0.039)	0.19
Time*Pre-pandemic mental distress			0.010 (0.042)	0.81
Time*Parental education			-0.035 (0.045)	0.44
Time*AN PGS			0.010 (0.033)	0.76
Time*Anxiety PGS			-0.002 (0.034)	0.95
Time*Depression PGS			0.049 (0.033)	0.14
Time*Neuroticism PGS			-0.000 (0.033)	0.99
**Random effects**				
ICC	0.621		0.622	
Marginal R^2/Conditional R^2	0.252/0.716		0.251/0.717	
	Model S5		Model S6	
**Main exposure associations**				
Quarantine frequency	0.035 (0.016)	**0.02**	0.076 (0.056)	0.17
Time	0.061 (0.035)	0.08	0.065 (0.035)	0.07
**Interactions**				
Time*Quarantine frequency				
Time*Sex			0.064 (0.034)	0.06
Time*Age			-0.040 (0.018)	**0.03**
Time*Pre-pandemic anxiety/depression			-0.121 (0.047)	**0.01**
Time*Pre-pandemic eating problems			0.010 (0.016)	0.53
Time*Pre-pandemic mental distress			0.034 (0.017)	**0.045**
Time*Parental education			-0.018 (0.020)	0.36
Time*AN PGS			0.015 (0.013)	0.25
Time*Anxiety PGS			0.047 (0.015)	**0.002**
Time*Depression PGS			-0.030 (0.013)	**0.03**
Time*Neuroticism PGS			-0.009 (0.015)	0.53
**Random effects**				
ICC	0.620		0.623	
Marginal R^2/Conditional R^2	0.252/0.716		0.255/0.719	

Supplementary models with pre-pandemic eating problems added to the models. Pre-pandemic mental distress was significant but did not pass multiple testing.

© 2024 Pettersen JH et al. *JAMA Network Open.*

eReferences

1. Sandanger I, Moum T, Ingebrigtsen G, Dalgard OS, Sørensen T, Bruusgaard D. Concordance between symptom screening and diagnostic procedure: the Hopkins Symptom Checklist-25 and the Composite International Diagnostic Interview I. *Soc Psychiatry Psychiatr Epidemiol*. 1998;33:345-354.
2. Nettelbladt P, Hansson L, Stefansson C-G, Borgquist L, Nordström G. Test characteristics of the Hopkins Symptom Check List-25 (HSCL-25) in Sweden, using the Present State Examination (PSE-9) as a caseness criterion. *Soc Psychiatry Psychiatr Epidemiol*. 1993;28:130-133.
3. Tambs K, Moum T. How well can a few questionnaire items indicate anxiety and depression? *Acta Psychiatr Scand*. 1993;87(5):364-367.
4. Government.no. Timeline: News from Norwegian Ministries about the Coronavirus disease Covid-19. Accessed November 14, 2023, https://www.regjeringen.no/en/topics/koronavirus-covid-19/timeline-for-news-from-norwegian-ministries-about-the-coronavirus-disease-covid-19/id2692402/
5. Hannigan LJ, Corfield, E.C., Askelund, A.D., Askeland, R.B., Hegemann, L., Jensen, P., Pettersen, J.H., Rayner, C., Ayorech, Z., Bakken, N.B., Wootton, R., Ask, H., Havdahl, A. phenotools: and R package to facilitate efficient and reproducible use of phenotypic data from MoBa and linked registry sources in the TSD environment. . 2023;doi:10.17605/OSF.IO/6G8BJ
6. Fairburn CG, Beglin SJ. Assessment of eating disorders: Interview or self-report questionnaire? *Int J Eat Disord*. 1994;16(4):363-370.
7. Choi SW, Mak TS, O'Reilly PF. Tutorial: a guide to performing polygenic risk score analyses. *Nat Protoc*. Sep 2020;15(9):2759-2772. doi:10.1038/s41596-020-0353-1
8. Purves KL, Coleman JR, Meier SM, et al. A major role for common genetic variation in anxiety disorders. *Mol Psychiatry*. 2020;25(12):3292-3303.
9. Howard DM, Adams MJ, Clarke T-K, et al. Genome-wide meta-analysis of depression identifies 102 independent variants and highlights the importance of the prefrontal brain regions. *Nat Neurosci*. 2019;22(3):343-352.
10. Bulik C, Duncan L, Breen G, Group PAW. The PGC Gwas Meta-Analysis of Anorexia Nervosa: SNP Heritability, Genetic Correlations, And Snp Results. *Eur Neuropsychopharmacol*. 2017;27:S360-S361.
11. Nagel M, Speed D, van der Sluis S, Østergaard SD. Genome-wide association study of the sensitivity to environmental stress and adversity neuroticism cluster. *Acta Psychiatr Scand*. May 2020;141(5):476-478. doi:10.1111/acps.13155
12. Choi SW, O'Reilly PF. PRSice-2: Polygenic Risk Score software for biobank-scale data. *Gigascience*. Jul 1 2019;8(7)doi:10.1093/gigascience/giz082
13. Coombes BJ, Ploner A, Bergen SE, Biernacka JM. A principal component approach to improve association testing with polygenic risk scores. *Genet Epidemiol*. 2020;44(7):676-686.

© 2024 Pettersen JH et al. *JAMA Network Open.*
