# Supplementary material for: COVID-19 Pandemic Quarantines and Mental Health Among Adolescents in Norway
Source: JAMA Netw Open. 2024 Jul 12;7(7):e2422189. doi: 10.1001/jamanetworkopen.2024.22189 (PMC11245726; doi:10.1001/jamanetworkopen.2024.22189)
Supplement: Supplement 2. — Data Sharing Statement [file jamanetwopen-e2422189-s002.pdf]

Data Sharing Statement

Pettersen. COVID-19 Pandemic Quarantines and Mental Health Among Adolescents in Norway. *JAMA Netw Open*. Published July 12, 2024.
doi:10.1001/jamanetworkopen.2024.22189

Data
Data available: No

Additional Information
Explanation for why data not available: Data from MoBa used in this study are managed by the national health register holders in Norway (Norwegian Institute of public health) and can be made available to researchers, provided approval from the Regional Committees for Medical and Health Research Ethics (REC), compliance with the EU General Data Protection Regulation (GDPR) and approval from the data owners. The consent given by the participants does not open for storage of data on an individual level in repositories or journals. Researchers who want access to data sets for replication should apply through helsedata.no. Access to data sets requires approval from REC in Norway and an agreement with MoBa.
